# Diffusion and Interaction Effects On Molecular Release Kinetics From Collapsed Microgels

**DOI:** 10.1021/acsapm.4c01150

**Published:** 2024-07-24

**Authors:** Sol Escañuela-Copado, José López-Molina, Matej Kanduč, Ana Belén Jódar-Reyes, María Tirado-Miranda, Delfi Bastos-González, José Manuel Peula-García, Irene Adroher-Benítez, Arturo Moncho-Jordá

**Affiliations:** † Grupo de Física de Fluidos y Biocoloides, Departamento de Física Aplicada, 16741 Universidad de Granada, 18071 Granada, Spain; ‡ 61790 Jožef Stefan Institute, SI-1000 Ljubljana, Slovenia; § Excellence Research Unit Modeling Nature (MNat), 117396 University of Granada, 18071 Granada, Spain; ∥ Departamento of Física Aplicada II, 16752 Universidad of Málaga, 29071 Málaga, Spain; ⊥ Instituto Carlos I de Física Teórica y Computacional, Facultad de Ciencias, 16741 Universidad de Granada, 18071 Granada, Spain

**Keywords:** DDFT, microgels, drug release, kinetics, diffusion, transport

## Abstract

The efficient transport of small molecules through dense hydrogel networks is crucial for various applications, including drug delivery, biosensing, catalysis, nanofiltration, water purification, and desalination. In dense polymer matrices, such as collapsed microgels, molecular transport follows the solution–diffusion principle: Molecules dissolve in the polymeric matrix and subsequently diffuse due to a concentration gradient. Employing dynamical density functional theory (DDFT), we investigate the nonequilibrium release kinetics of nonionic subnanometer-sized molecules from a microgel particle, using parameters derived from prior molecular simulations of a thermoresponsive hydrogel. The kinetics is primarily governed by the microgel radius and two intensive parameters: the diffusion coefficient and solvation free energy of the molecule. Our results reveal two limiting regimes: a diffusion-limited regime for large, slowly diffusing, and poorly soluble molecules within the hydrogel; and a reaction-limited regime for small, rapidly diffusing, and highly soluble molecules. These principles allow us to derive an analytical equation for release time, demonstrating excellent quantitative agreement with the DDFT results—a valuable and straightforward tool for predicting release kinetics from microgels.

## Graphical abstract

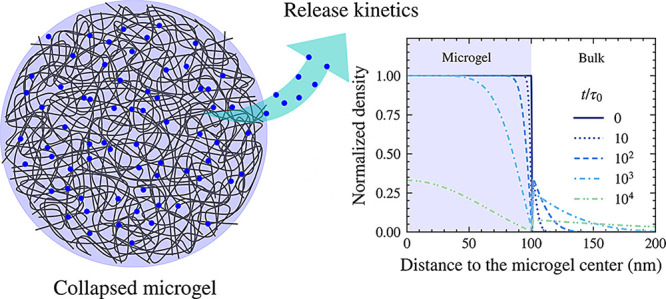

## 1 Introduction

Microgels, cross-linked polymer networks of sizes ranging from hundreds of nanometers to around a micrometer, remain an active area of research in disciplines like nanotechnology,^1^ materials science,^2^ environmental sciences,^3^ and biomedical sciences.^4^ These colloidal particles are recognized for their stimuli-responsive behavior, which is characterized by reversible volume phase transitions in response to environmental stimuli such as changes in temperature,^5^ pH,^6^ ionic strength,^7^ or solvent properties.^8^ This unique adaptability and functional versatility enable microgels to serve in a wide range of applications, from surface coating^9^ to optoelectronic switches.^10^ In particular, their responsiveness to environmental changes further broadens their utility in medicine. They can act as effective containers for various molecules, such as proteins, polysaccharides, enzymes, nucleic acids and drugs, providing a foundation for advancements in drug accumulation, release, and targeted delivery.^11–13^ Such capabilities underscore the significant promise of microgels in driving the evolution of drug delivery systems and further developments in nanotechnology and nanomedicine.^14^

Poly­(*N*-isopropylacrylamide) (PNIPAM) is one of the most extensively studied thermoresponsive polymers, mainly because it exhibits a volume transition in water from a swollen to collapsed state at temperatures closely approximating the human body temperature.^15,16^ Given its versatility, PNIPAM microgels have served as fundamental model systems, paving the way for numerous advancements in the field of soft responsive materials.^17^

The control over the uptake and release kinetics of molecules from nanoparticulate systems is crucial for realizing their full potential in a range of applications. For targeted drug delivery, the precise modulation of the kinetics is key to achieving desired therapeutic outcomes.^18,19^ Properly tuned release rates can ensure consistent drug concentrations in the systemic circulation, while efficient uptake directly impacts drug loading capacity, influencing overall therapeutic efficacy. In industrial contexts, control over uptake and release kinetics can directly influence process efficiency in catalysis or chemical separation, where the kinetics can dictate reaction rates and separation performance.^20^ When utilized as nanoreactors, the responsive network structure of microgel particles allows for modulation of the permeability of reactants, thereby determining the reaction rate.^17,21^ Therefore, understanding and manipulating these kinetics is a central aspect of nanocarrier optimization, providing a solid foundation for more predictable and reliable system performance across various applications. This understanding is essential not only for the practical implementation of these systems but also for the development of accurate theoretical models.

In most of the aforementioned applications, the hydrogel matrix exhibits high density.^22,23^ In such regimes, the interplay between the molecule and the polymer matrix, as well as its transport through a densely crowded environment, becomes notably complex owing to polymer–water interactions and obstruction effects.^24^ Many traditional size-exclusion theories fail to accurately predict the solvation free energy and, consequently, the resulting partition ratio in these scenarios. They often predict lower values for the concentration ratio of cargo within the microgel compared to its bulk concentration. Notably, the partition ratio of hydrophobic molecules (such as drugs) within the microgel can be significantly higher, sometimes exceeding theoretical predictions by several orders of magnitude.^25,26^ This phenomenon has been further corroborated by atomistic computer simulations, which have shown that the calculated solvation free energies (i.e., transfer free energies from the bulk solution to the interior of a collapsed polymer gel) can be considerably negative.^27–29^ This suggests a tendency for molecules to remain inside the gel.

The diffusive transport of cargo molecules across a collapsed microgel is also a complex process,^30^ intrinsically different from diffusion in simple liquids and dilute systems.^31–33^ Atomistic computer simulations offer valuable insights into the behavior of molecules within dense polymer networks at a microscopic scale. Studies reveal various distributions of water molecules—either evenly dispersed^34^ or forming clusters.^35^ For hydrophobic collapsed PNIPAM networks, a combination of atomistic simulations^28,36^ and experimental data^37^ suggests that water molecules form disconnected, fractal-like clusters absorbed within voids created by polymeric chains. In these dense systems, where free-volume elements within the polymer network emerge due to thermal fluctuations at a similar time scale as molecule movements, the collapsed microgel functions as a nonporous membrane. Molecular transport in collapsed microgels follows a solution–diffusion mechanism.^38^ Here, cargo molecules dissolve within the densely packed polymeric matrix and subsequently diffuse due to concentration gradients. At this level of description, these dense microgels can often be regarded as a continuum.

In this work, we make use of dynamical density functional theory (DDFT) to model the release of molecules previously encapsulated inside a collapsed microgel.^39–41^ DDFT is a theoretical framework that has evolved as an effective tool for studying the dynamics of many-body systems in the field of condensed matter physics and soft matter science. This technique extends classical DFT to incorporate time-dependent phenomena, thereby enabling the investigation of out-of-equilibrium states.

It is important to emphasize that DDFT serves as a coarse-grained, macroscopic framework for describing nonequilibrium processes. In the context of drug release, DDFT offers insights into the spatiotemporal evolution of the density profile of molecules, ρ­(**r**, *t*), throughout the release process. The method takes into account the actual sterically obstructed diffusion coefficient of the molecules inside the microgel. Furthermore, it incorporates the effects of both the microgel–molecule and molecule–molecule interactions. DDFT has been successfully applied to similar problems, namely, the prediction of protein adsorption into nanoparticles^42,43^ and the encapsulation/release kinetics of neutral and charged molecules in hollow microgel particles.^29,44,45^

In the literature, various coarse-grained free energy functionals describe a cross-linked polymer network of microgels as a quenched mobile random matrix composed of hard or soft core particles. Within this matrix, molecules diffuse under the influence of an effective external potential.^46,47^ However, in this work, we construct our DDFT framework following a more macroscopic description by considering the effective diffusion coefficient, D*, and the transfer free energy, Δ*G*, obtained from previous atomistic molecular dynamics simulations of different neutral molecules within collapsed PNIPAM polymer networks.^27,28,36^ Thus, although our DDFT approach is intrinsically macroscopic, it incorporates microscopic details regarding the complex interactions of the molecules with the polymer network, which may become extremely important in the case of collapsed networks.

The novelty of this work is two-fold. First, we demonstrate that the time evolution of the fraction of released molecules can be effectively scaled into a master curve that only depends on the half-release time, τ_1/2_. This finding suggests a potential universal behavior in the release dynamics of different molecules, indicating that τ_1/2_ alone is sufficient to fully describe the release kinetics of any molecule. Second, we derive an analytical expression for the half-release time, incorporating the key parameters that rule the nonequilibrium kinetics of the release process, namely, the microgel radius, the solvation free energy of the cargo molecules, and their diffusion coefficients. This result not only provides a conceptual framework for understanding release kinetics but also facilitates preliminary predictions that can guide subsequent experimental work. To the best of our knowledge, such a simple yet powerful mathematical expression has not been reported in the existing literature for collapsed microgels.

This paper is organized as follows. After this Introduction, we describe our Theoretical Methods, detailing the model for cargo release from a collapsed microgel and explaining the theoretical framework applied in our study. In the following Section 3, we present our findings, exploring the release kinetics of molecular cargo from collapsed microgels, solving the DDFT equation. We analyze the time-dependent density profile of cargo molecules as they diffuse through the polymer network and discuss the time evolution of the fraction of released molecules. This is followed by a comprehensive study of the influence of the diffusion coefficient and the molecule–microgel interaction free energy on release dynamics. We also analyze the role of microgel size in the release behavior. One of the main contributions of this study is to show that solving the stochastic differential equation for mean-first passage time allows the full set of results to be gathered into a single analytical expression that correctly describes the release kinetics for any particular situation. In the Conclusions section, we summarize our primary findings and discuss their broader implications.

## 2 Theoretical Methods

### 2.1 Model for Cargo Release from a Collapsed Microgel

To theoretically investigate the release of molecular cargo from a collapsed microgel, we model our system by considering a single spherical microgel particle of radius *b* immersed in an aqueous solution, treated as a uniform background. Figure 1 shows a schematic illustration of the microgel particle. Although the interior of the particle exhibits localized fluctuations in polymer density, it is still possible to define an average local polymer volume fraction, ϕ_p_. For a collapsed microgel, ϕ_p_ is roughly constant inside the microgel and abruptly declines to zero at the external interface. This radial dependence can be approximated as ϕ_p_(*r*) = ϕ_p_^0^ *f*(*r*), with

$$f(r)=\frac{1}{2}[1-\mathrm{erf}(2(r-b-\delta )/\delta )]$$ 1

where *r* represents the distance to the microgel center, erf­(*x*) is the error function, 2δ is the thickness of the interface, and ϕ_p_^0^ ≈ 0.5 is the accepted value of polymer volume fraction inside a collapsed microgel.^48^ This functional dependence leads to the required polymer distribution: it is roughly uniform inside the microgel with ϕ_p_(*r*) ≈ ϕ_p_^0^ for *r* < *b* and decays to zero near the external interface, spanning from *r* = *b* to *r* = *b* + 2δ. For collapsed microgels, the experimental data report a very sharp interface, with a value for the interface half-thickness of about δ = 1 nm.^48^ We will set the system temperature at 340 K, well above the lower critical solution temperature of PNIPAM, where microgel particles, composed of this thermoresponsive polymer, are in their collapsed state. This relatively high temperature was chosen to utilize parameters from a previous simulation study,^28^ where the elevated temperature facilitated the dynamics and sampling.

**Figure 1:**
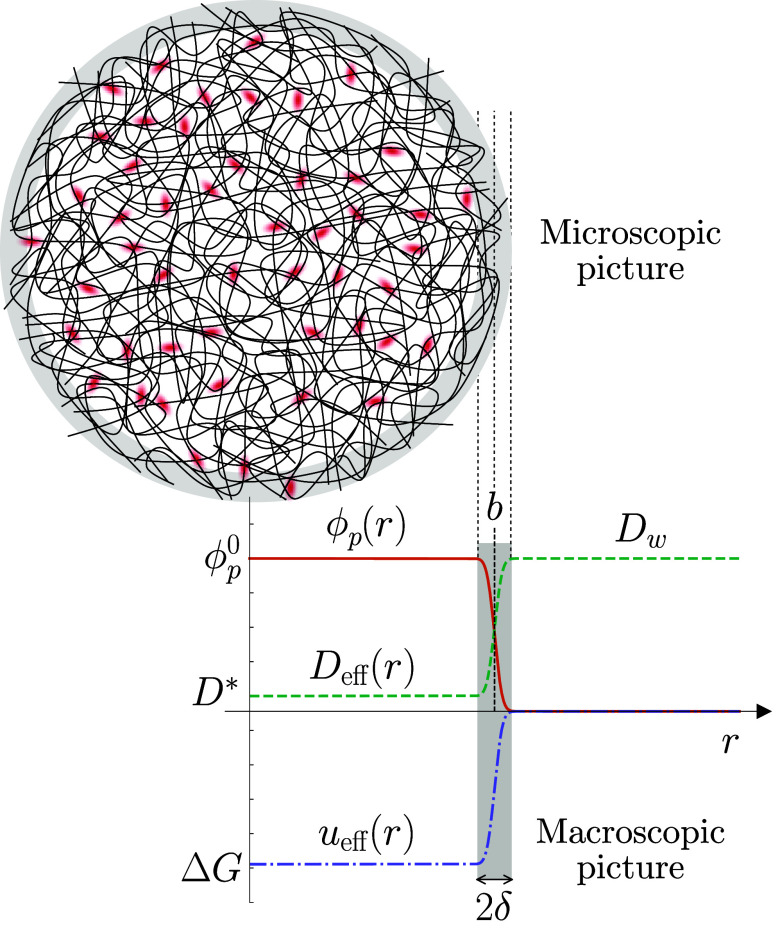
Representation of a collapsed microgel of radius *b* with a narrow interface of width 2δ ≪ *b* carrying cargo molecules. The plot illustrates the radial dependence of the polymer volume fraction (ϕ_p_(*r*)), the effective diffusion coefficient of the cargo molecule (*D*_eff_(*r*)), which switches from D* inside the microgel to *D*_w_ in bulk, and the effective microgel–molecule interaction (*u*_eff_(*r*)), which is given by Δ*G* inside the microgel and decays to 0 at the interface with the bulk.

In the initial stage, the microgel is loaded with a certain amount of cargo molecules. Our model does not specify a particular molecule, thus making it broadly applicable to a wide range of nonionic encapsulated entities, including chemical reactants, biomolecules, and pharmaceutical compounds. It should be noted that charged molecules may involve long-range electrostatic interactions, leading to a very different behavior. As corroborated by a recent simulation study,^28^ the size and shape of the nonionic molecule contribute to its diffusion coefficient. We proceed under the assumption of a generic cargo molecule, characterized by a hydrodynamic (Stokes) radius *a*_w_. This radius is determined from the diffusion coefficient of the molecule in water, *D*_w_, using the Stokes–Einstein relation,

$$a_{w}=\frac{k_{B}T}{6\pi \eta D_{w}}$$ 2

Here, *T* is the absolute temperature, *k*_B_ is the Boltzmann constant, and η = 4.206 × 10^–4^ Pa s is the viscosity of water at *T* = 340 K.

The two key parameters that strongly control the release kinetics are the effective diffusion coefficient of the cargo molecule inside the microgel, *D*_eff_, and the effective interaction between the molecule and the collapsed polymeric network, *u*_eff_. On the one hand, *D*_eff_ determines the time the molecule needs to diffuse from the initial position inside the microgel to its external interface. This time scales as τ ∼ *l* ^2^/*D*_eff_, where *l* is a characteristic length of the system, included for dimensional consistency and related to the size of the microgel. On the other hand, *u*_eff_ represents the free energy difference that the molecule must overcome in order to escape to the bulk solution. According to the Arrhenius law,^49^ the typical time implied by the molecule to surpass this energy barrier is expected to scale as exp­(−*u*_eff_/(*k*_B_ *T*)). Since both quantities describe local properties of the polymer matrix, they depend on the distance to the center of the microgel, *r*. Given the great importance of both functions, we discuss them in detail in the following two sections.

#### 2.1.1 Effective Diffusion Coefficient

The effective diffusion coefficient varies from inside the microgel, denoted by D*, to the corresponding value outside (i.e., in bulk water), given by *D*_w_. The switch between these two values at the interface is modeled as

$$D_{\mathrm{eff}}(r)=D_{w}+(D^{*}-D_{w})f(r)$$ 3

where *f*(*r*) is given by eq 1. This dependence is illustrated in Figure 1, featuring a sharp increase at the interface.

A key feature of collapsed hydrogels is the fluctuating free-volumes formed between the polymer chains, which allow the molecules to diffuse through the solution–diffusion mechanism.^38,50^ Experiments^51,52^ and computer simulations^31,32,53^ on these systems have demonstrated that molecular diffusion is significantly influenced by this characteristic of the polymer network. The diffusion coefficient within such dense polymer networks can be orders of magnitude lower than in bulk water, D*/*D*_w_ ≈ 10^–4^–10^–2^, as shown by other studies.^36,54^ In addition, D* decreases exponentially with the molecule Stokes radius^27^ ^,^ ^28^:

$$D^{*}=D_{0}e^{-a_{w}/\lambda }$$ 4

where *D*_0_ is a reference diffusion constant, and λ is a characteristic length scale that exclusively depends on the shape of the diffusing molecule. Schweizer and co-workers^55,56^ offered a theoretical explanation for the exponential relationship between D* and the size of the molecule, grounded in the coupled dynamics in dense liquids.

It is important to emphasize that D* for nonionic molecules is not affected by the polymer-molecule affinity.^28^ For instance, the value of D* is the same for polar and nonpolar cargo molecules, provided that the size and shape of both molecules are the same in both cases. This is a singular feature of collapsed polymer networks, for which the diffusion of the cargo only depends on its geometrical properties through the steric exclusion effects induced by the polymer chains.^27,28^ The reason for that relies on the fact that, during a jump of the molecule inside such a dense polymer network, the coordination of its solvation shell and the number of contacts with the surrounding polymers are not significantly altered, so the free energy change between the old and the new position is very small compared to obstruction effects caused by the local trapping, which only depends on the size and shape of the molecule.^27,28^

#### 2.1.2 Relative Shape Anisotropy

The stringent constraints imposed by a dense polymer matrix on the motion of cargo molecules lead to preferential diffusion directions dictated by the geometry of the cargo molecule. Employing a simple qualitative geometric criterion, molecules can be categorized into linear, planar, and spherical groups, as demonstrated in a previous atomistic study.^28^ This classification was employed to examine the dynamics of the diffusion coefficient and the depth of potential barriers. It was revealed that, for molecules with the same Stokes radii (see eq 2), linear molecules have the most favorable diffusion, as their shape allows easy penetration through narrow channels between polymer chains. Planar molecules, displaying intermediate diffusion efficiency, use their shape to diffuse in a sideways manner. Conversely, spherical molecules encounter the greatest difficulty during diffusion. Consequently, for a given Stokes radius, the diffusion coefficient follows the order *D*_linear_^*^ > *D*_planar_^*^ > *D*_spherical_^*^.

In this work, we adopt a slightly different approach using quantitative criteria to determine diffusion coefficients based on molecular shape. We compute the cargo radius of gyration tensor, **G**, a method frequently used to characterize the shape of polymers.^57^ Performing the diagonalization of the gyration tensor yields three eigenvalues, (α_1_, α_2_, and α_3_). This intuitively evokes the representation of an ellipsoidal shape, where the eigenvalues correspond to the semiaxes of the ellipsoid. To identify the molecule shape, we calculate a shape descriptor derived from the gyration tensor, called relative shape anisotropy^58,59^ :

$$\kappa \;≡\;\frac{3}{2}\frac{\mathrm{Tr}G^{2}}{(\mathrm{Tr}G{)}^{2}}=1-3\frac{\alpha_{1}\alpha_{2}+\alpha_{1}\alpha_{3}+\alpha_{2}\alpha_{3}}{(\alpha_{1}+\alpha_{2}+\alpha_{3}{)}^{2}}$$ 5

which can range from 0 to 1. A linear arrangement of atoms corresponds to κ = 1, a regular planar geometry corresponds to κ = 0.25, and structures of tetrahedral symmetry or higher, such as spheres, correspond to κ = 0.^58^ We computed the relative shape anisotropy for each molecule in Table 2, classifying those within a tolerance of Δκ = 0.1.

Figure 2 plots the diffusion coefficient prefactor, *D*_0_, and decay length, λ, from eq 4, as functions of the relative shape anisotropy, κ. The diffusion coefficients were taken from a previous simulation work of a collapsed PNIPAM hydrogel.^28^ Molecules are categorized into spherical, planar, or linear groups based on their κ values, and within each group, average values ⟨*D*_0_⟩ and ⟨λ⟩ are calculated. The data are then fitted using the following functions:

$$\lambda =m_{1}\kappa +n_{1}$$ 6

$$D_{0}=n_{2}e^{m_{2}\kappa }$$ 7

**Figure 2:**
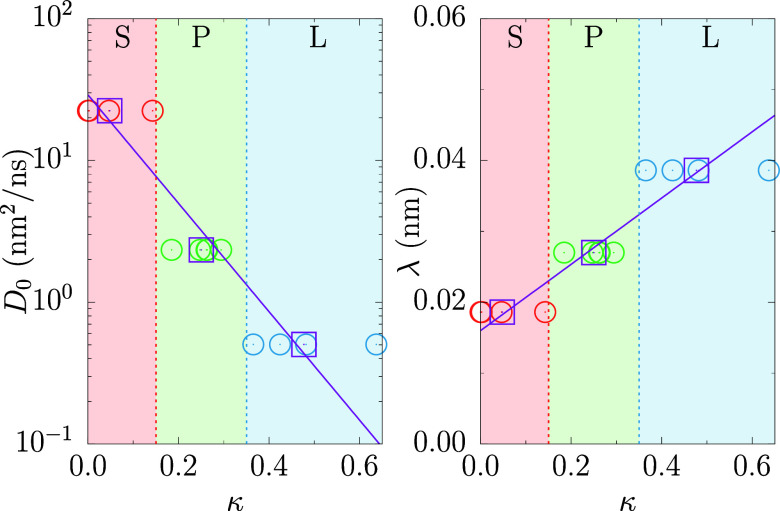
Diffusion coefficient prefactor (*D*_0_) and decay length (λ) as a function of relative shape anisotropy (κ). Each panel is divided into three sections corresponding, from left to right, to the spherical (S), planar (P), and linear (L) shapes of the molecules, respectively.

The left panel of Figure 2, employing a linear-log scale, reveals an exponential relationship between *D*_0_ and κ, as outlined in eq 7. The right panel displays a linear correlation between λ and κ, as described by eq 6. The values of the fitting parameters for each plot are compiled in Table 1. These parameters allow us to compute *D*_0_ and λ and, therefore, the value of D* for every nonionic molecule by using eq 4, just by computing the corresponding shape anisotropy, κ (Table 2).

**Table 1:** Numerical Values for the Fitting Parameters Obtained From the Fits in Figure 2 ^a^

fitting parameter	value	standard error
*m*_1_ (nm)	0.047	0.003
*n*_1_ (nm)	0.0160	0.0009
*m*_2_	–8.8	1.3
*n*_2_ (nm^2^/ns)	3.37	0.39

a Parameters (*m*_1_, *n*_1_) are derived from the linear fit of λ as a function of κ. Parameters (*m*_2_, *n*_2_) result from the linear-log fit of *D*_0_ as a function of κ.

**Table 2:** Transfer Free Energies and Diffusion Coefficients, Taken from a Previous Simulation Work,^28^ Which are Investigated in This Study^a^

molecule	symbol	κ	βΔ*G*	D*/*D*_w_ × 10^4^
Linear				
Nitrophenol	NP	0.365102	–7.90	1.5
Pentane	Pe	0.481603	–6.22	4
Pentanol	PeOH	0.424164	–4.44	3.2
				
Planar				
Nitrobenzene	NB	0.294118	–7.54	3.6
Phenol	Ph	0.263224	–6.19	2.4
Benzene	B	0.247294	–4.88	3.7
Propanol	PrOH	0.261749	–2.07	6.3
Methanol	MeOH	0.184735	–0.36	27
				
Spherical				
Tetrachloromethane	CCl_4_	0.046486	–8.75	1.12
Neopentane	NPe	0.000002	–8.43	0.77
Ethane	Et	0.142804	–3.44	17
Methane	Me	0.002383	–1.88	71
Argon	Ar	0.000000	–1.51	135
Neon	Ne	0.000000	–0.20	810
Helium	He	0.000000	0.19	1970

a Molecules have been classified based on the relative shape anisotropy, as defined by eq 5.

#### 2.1.3 Effective Molecule–Microgel Interaction

Transferring the molecule from the bulk solution (water phase) into the microgel (polymer phase) is characterized by the free energy difference, Δ*G*. In a similar fashion as we modeled ϕ_p_(*r*) and D*­(*r*), we assume the radial variation of the effective interaction as *u*_eff_(*r*) = Δ*Gf*(*r*). In contrast to the diffusion coefficient, Δ*G* depends not only on the size and shape of the molecule but also on its chemical nature, particularly its polarity. Thus, the molecule’s affinity to the polymer matrix is strongly influenced by its hydrophobic or hydrophilic nature. In a simple phenomenological approach, this energetic contribution scales very well with the molecule’s surface area and can be written as^28,36^

$$\Delta G=\Delta G_{0}+\gamma_{0}4\pi a_{\mathrm{AS}}^{2}$$ 8

In the second term, 4π*a*_AS_^2^ is the solvent-accessible surface area of the molecule, expressed with an equivalent spherical radius, *a*_AS_. It should be noted that *a*_AS_ is strongly linked to the Stokes radius, and for a wide range of diverse molecules, it can be expressed as *a*_AS_ = *a*_w_ + 0.233 nm.^28^ The coefficient γ_0_ can be interpreted as the disparity between the surface tensions of the molecule with the polymer and the molecule with water. The first term in eq 8, Δ*G*_0_, reflects the chemical nature of the molecule, correlating with the number of polar groups present within it.

We emphasize that for a specific molecule, the values of D* and Δ*G* provided, respectively, by eqs 4 and 8 are not mutually independent. Indeed, varying the size or shape of the molecule will affect both parameters. Therefore, they are interconnected for each microgel–molecule pair. However, in this work, we systematically explore different values of these two parameters, treating them as independent to understand the role of each in the release kinetics.

### 2.2 Dynamical Density Functional Theory

To investigate the nonequilibrium diffusive release of cargo molecules, we make use of classical DDFT.^39–41,60^ DDFT is a theoretical framework for the time evolution of the one-body density of a fluid. It extends the original framework of the DFT, which was designed for equilibrium systems,^61–64^ to address nonequilibrium cases. This method can be used to describe how an initial nonequilibrium density profile of molecules evolves in time in the presence of an external potential exerted by the microgel. In particular, it provides the local concentration of the cargo on position **r** at time *t* during the release process, ρ_c_(**r**, *t*), starting from an initial distribution, ρ_c_(**r**,0).

In contrast to the usually employed diffusion equation, DDFT has three important improvements. First, the diffusion of the cargo takes into account the interaction of the molecule with the external field exerted by the polymer network of the microgel, *u*_eff_(*r*). Second, the method also considers the position dependence of the diffusion coefficient, *D*_eff_(*r*). Indeed, this is precisely the case when the molecule travels from the interior of the microgel to the bulk solution, where the diffusion constant changes from D* to *D*_w_. Third, DDFT also incorporates the effect of the molecule–molecule interactions by means of the corresponding excess free energy.

Within the DDFT approach, the cargo concentration obeys the following continuity equation^39^ ^,^ ^60^:

$$\frac{\partial \rho_{c}(r,t)}{\partial t}=-\nabla \cdot J_{c}(r,t)$$ 9

where *J*_c_(**r**, *t*) denotes the space and time-dependent diffusive current density, given by

$$J_{c}=-D_{c}(r)[\nabla \rho_{c}+\rho_{c}\beta \nabla (u_{\mathrm{eff}}(r)+\mu_{c}^{\mathrm{ex}}(r,t))]$$ 10

where β = 1/(*k*_B_ *T*). The excess nonequilibrium chemical potential, μ_c_^ex^(**r**, *t*), gathers the effect of molecule–molecule interactions. In general, it is obtained from the functional derivative of the equilibrium excess free energy of the molecules.^61,62^ Here, we use a simple mean-field prescription to account for excluded-volume interactions, given by the Carnahan–Starling expression^61^:

$$\beta \mu_{c}^{\mathrm{ex}}(r,t)=\phi_{c}(r,t)\frac{8-9\phi_{c}(r,t)+3\phi_{c}(r,t{)}^{2}}{[1-\phi_{c}(r,t){]}^{3}}$$ 11

where $\phi_{c}(r,t)=\frac{4}{3}\pi a_{w}^{3}\rho_{c}(r,t)$ is the local volume fraction of molecules. We remark here that this model does not introduce attraction between molecules, which may lead to phase separation of the diffusing molecules inside the microgel. For weak attractions, this effect can be effectively included in the model by simply renormalizing Δ*G*.^65,66^

Equations 9 and 10 have to be solved numerically for spherical symmetry. Three boundary conditions are required. The first one establishes the initial distribution of cargo molecules. In this regard, there are two possibilities for the passive release experiments. One possibility is to initially load the microgel with the cargo molecules until equilibrium is reached. After that, the supernatant is replaced by fresh solvent (e.g., pure water) so that the encapsulated molecules are in a nonequilibrium state, and the release process begins. The other option is to use microgels in a lyophilized and collapsed state with the molecules trapped inside, which is a common procedure in many release experiments. When these lyophilized microgels are rehydrated by immersion in a solvent (e.g., water), the release process begins. In both scenarios, the cargo molecules are initially uniformly distributed inside the microgel particle, whereas their concentration outside the microgel is zero,

$$\rho_{c}(r,t=0)=\{\begin{array}{cc} \rho_{c}^{0} & r\leq b\\ 0 & r>b \end{array}$$ 12

Second, the diffusive current density in the center of the microgel must be zero due to the spherical symmetry of the system,

$$J_{c}(r=0,t)=0,\forall t,$$ 13

Finally, for a very diluted suspension of microgel particles, we can assume that the volume of the bulk solution surrounding the microgel is very large such that the cargo concentration far away from the microgel is zero. This implies the following absorbing boundary condition:

$$\rho_{c}(r\to \infty ,t)=0,\forall t,$$ 14

For practical reasons, we consider that a distance *R* = 20*b* is large enough to apply this boundary condition such that ρ_c_(*r* = *R*, *t*) = 0.

We emphasize again that, although the DDFT framework provides a macroscopic description for the diffusive release of cargo molecules, it effectively encapsulates microscopic details linked to the complex diffusion and interactions of the cargo molecule within the crowded environment of a collapsed microgel. Namely, our macroscopic description considers the microscopic complexity through parameters D* and Δ*G* obtained from atomistic MD simulations (see eqs 4 and 8).

In our calculations, cargo molecules are assumed to be released to the bulk solution when they reach the outer interface of the microgel, that is, when *r* > *b* + 2δ. With this prescription, the fraction of released cargo is given by

$$f_{\mathrm{rel}}(t)=1-\frac{4\pi }{N_{0}}\int_{0}^{b+2\delta }r^{2}\rho_{c}(r,t)dr$$ 15

where *N*_0_ is the number of molecules encapsulated inside the microgel in the initial state, $N_{0}=\frac{4}{3}\pi b^{3}\rho_{c}^{0}$ . We define the half-release time, τ_1/2_, as the time required to release 50% of the encapsulated cargo, i.e., *f*_rel_(τ_1/2_) = 0.5. As we will see later, τ_1/2_ holds immense significance as it consolidates the most pertinent details regarding the release kinetics into a singular parameter. In fact, the knowledge of the scaling τ_1/2_ with D* and Δ*G* is especially of vital help to estimate extremely slow release kinetics where the integration of the DDFT equation can involve prohibitively long calculations.

In addition to τ_1/2_, we can define the mean release time of the encapsulated molecules, given by

$$\mathrm{\tau ̅}=\int_{0}^{\infty }t\left(\frac{df_{\mathrm{rel}}}{dt}\right)dt$$ 16

To solve the time-dependent DDFT differential equation, distances were scaled by *l*_0_ = 1 nm and time by τ_0_ = *l*_0_^2^/*D*_w_. In order to shorten the computation time of the numerical resolution, a nonuniform spatial grid was used to sample the distance *r*. We used a very narrow grid size of Δ*r*_min_ = 0.02*l*_0_ at the microgel interface, where the gradients of *D*_eff_(*r*) and *u*_eff_(*r*) are larger, whereas thicker size intervals Δ*r*_min_ = 0.5*l*_0_ were employed in the regions inside and outside the microgels. On the other hand, a time step of Δ*t* = 10^–4^τ_0_ was used in all the calculations. This value is smaller than (Δ*r*_min_)^2^/(2*D*_0_), thus preventing the occurrence of nonphysical sawtooth instabilities.

## 3 Results and Discussion

In this section, we make use of the DDFT theoretical framework described above to study the release kinetics of the molecules encapsulated within the collapsed microgel. This technique provides the time evolution of the cargo’s density profiles, enabling us to determine the fraction of released molecules and the characteristic release time. In all the cases studied here, the initial encapsulated cargo concentration contained inside the microgel is $\rho_{c}^{0}=0.01\;M$ .

### 3.1 Spatiotemporal Evolution of Cargo Molecules

Figure 3 shows the time evolution of the local density of cargo molecules normalized by the initial density inside the microgel, ρ_c_(*r*, *t*)/ρ_c_^0^, for two molecular sizes and three molecule–microgel interaction free energies, covering attractive, neutral, and repulsive polymer networks (βΔ*G* = −3, 0, and 3, respectively). We consider the particular case of spherical molecules (κ = 0), although similar results are found for other shapes. The results are organized so that each column represents a specific molecule size. From these graphs, it becomes clear that the larger molecules are released at a slower rate than their smaller counterparts. This inverse relation between the molecular size and the release rate can be attributed to the exponential decay of the diffusion coefficient with the molecule size, *a*_w_.

**Figure 3:**
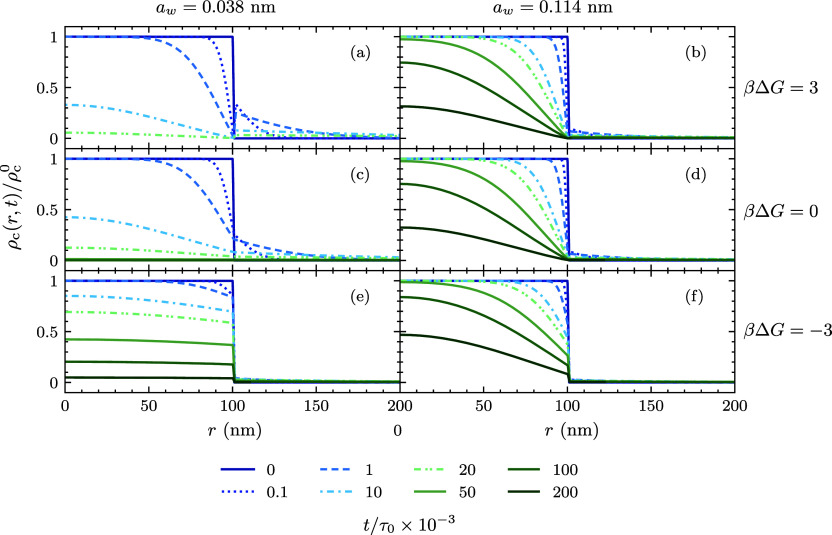
Spatial evolution of the density of cargo molecules normalized by their initial density inside the microgel; different lines represent different snapshots in time. Each column depicts the results for a given diffusion coefficient (or molecular size). In panels (a), (c), and (e), the Stokes radius corresponds to that of helium, that is, *a*_w_ = 0.038 nm. Additionally, in panels (b), (d), and (f), the cargo molecular size is restricted to the methane Stokes radius, which is *a*_w_ = 0.114 nm. Every row of panels represents a specific value of the potential barrier βΔ*G*: (a) and (b) to βΔ*G* = 3, (c) and (d) to βΔ*G* = 0, and (e) and (f) to βΔ*G* = −3. In all cases, the geometry of the molecule is spherical, ρ_c_^0^ = 0.01 M, and δ = 1 nm.

Figure 3 also shows the influence of various free energy values on the release process. Each row of panels corresponds to a specific value of βΔ*G*. We observe that attractive microgels remarkably slow down the release process, which can be observed in the difference between concentration profiles over time when comparing βΔ*G* = −3 to other potential barriers. Important differences are observed between the time-dependent density profiles in repulsive and attractive polymer networks. For repulsive networks, such as βΔ*G* = +3, the cargo molecules are energetically expelled from the microgel, leading to a dynamic depletion of molecules near the microgel interface (see Figure 3a,b). However, when comparing these profiles with those from the no potential barrier case (βΔ*G* = 0), we observe no significant differences. This indicates that a positive energy barrier has minimal impact on the release dynamics. Conversely, for attractive networks (βΔ*G* = −3), molecules are retained inside the microgel because they need to overcome a free-energy step-edge barrier to escape from the interior of the microgel to the bulk solution, giving rise to a more uniform distribution of molecules, especially for small cargo molecules, as seen in Figure 3e.

### 3.2 Time Evolution of the Fraction of Released Molecules

Integrating the density profiles ρ_c_(*r*, *t*) in eq 15 leads to the time-dependent fraction of release cargo, *f*_rel_(*t*). In Figure 4, we depict *f*_rel_(*t*) for a specific set of molecules, covering small and large sizes and different molecule–microgel interaction free energies. As observed, *f*_rel_(*t*) exhibits a profile consistent with a cumulative distribution function. As previously mentioned, a very important quantity that characterizes the release process is the half-release time, τ_1/2_, defined as the time at which 50% of the molecules have been released. Notably, while all the curves exhibit analogous profiles, the primary variance among them is attributed to the τ_1/2_ value, arising from the particular release dynamics of each molecule. Indeed, the curve corresponding to the diffusive release of large-sized molecules that are strongly attracted to the microgel is shifted to longer times compared to small molecules weakly attracted to the microgel because the former ones diffuse slower and also need to surpass a higher free energy barrier to reach the bulk solution.

**Figure 4:**
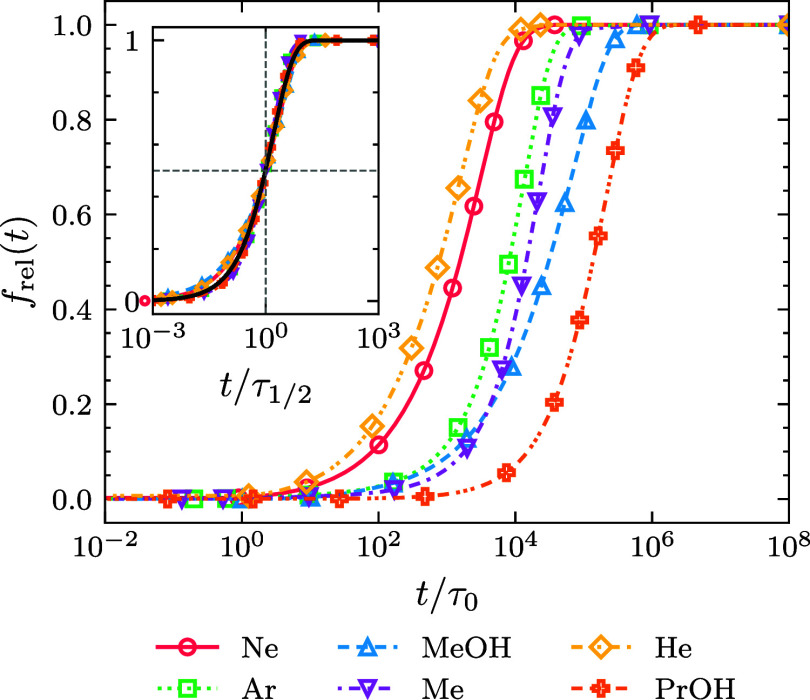
Time evolution of the fraction of released cargo for different molecules. The inset represents the same data set, with the time axis normalized by the half-release time. A reference grid helps emphasize how all the renormalized curves align at (1, 0.5). In all cases, the used parameters are ρ_c_^0^ = 0.01 M, δ = 1 nm, and *b* = 50 nm. The black solid line shows the fit provided by eq 17.

Normalizing the time by the respective half-release times for each molecule allows all these curves to converge into a master curve, as shown in the inset of Figure 4. The overlap of all of the release profiles upon this normalization suggests a potential universal feature of the release process, which is scalable across different molecular types. In addition, this scaling demonstrates that τ_1/2_ is enough to fully predict the release kinetics of any molecule. The percentage of released cargo molecules of various shapes and sizes can be described by a common Weibull cumulative distribution function^67,68^

$$f_{\mathrm{rel}}(t)=1-\exp[-(\chi t/\tau_{1/2}{)}^{\nu }]$$ 17

where χ and ν are the fitting parameters controlling the shape of the function, with values χ = 0.64 ± 0.04 and ν = 0.79 ± 0.13, respectively, as determined from fitting the DDFT results. From this cumulative distribution, the mean release time of the molecules can be calculated using eq 16, resulting in

$$\overset{-}{\tau }=1.787\tau_{1/2}$$ 18

### 3.3 Effect of the Diffusion Coefficient and Molecule–Microgel Interaction Free Energy

Recognizing τ_1/2_ as the inherent time scale of the release, it becomes imperative to thoroughly explore its dependency on D* and Δ*G* as the main material parameters of our system. The goal is to find an analytical expression τ_1/2_(D*, Δ*G*) capable of universally characterizing the release kinetics of any nonionic molecule from collapsed microgels.

Figure 5 illustrates how the half-release time, derived from solving the DDFT equation, correlates with the effective diffusion coefficient D* for different Δ*G* values, including both repulsive and attractive interactions. For molecules with a small diffusion coefficient, the release of cargo is primarily governed by diffusion. This observation aligns with well-established diffusion principles of Brownian particles, which state that the mean time to travel a fixed distance is inversely proportional to the diffusion coefficient, i.e., τ ∼ 1/D*. We emphasize that, in this diffusion-limited regime, the effect of Δ*G* is negligible because the release kinetics are completely controlled by the time the molecules need to diffuse to the external interface of the microgel. The time to surpass the microgel interface is only a minor correction in this case. Consequently, all curves with different Δ*G* collapse onto a common form in this regime. The use of a normalized release time τ_1/2_/τ_0_ enhances the visibility of this collapse since it removes the dependence of the half-release time on *D*_w_.

**Figure 5:**
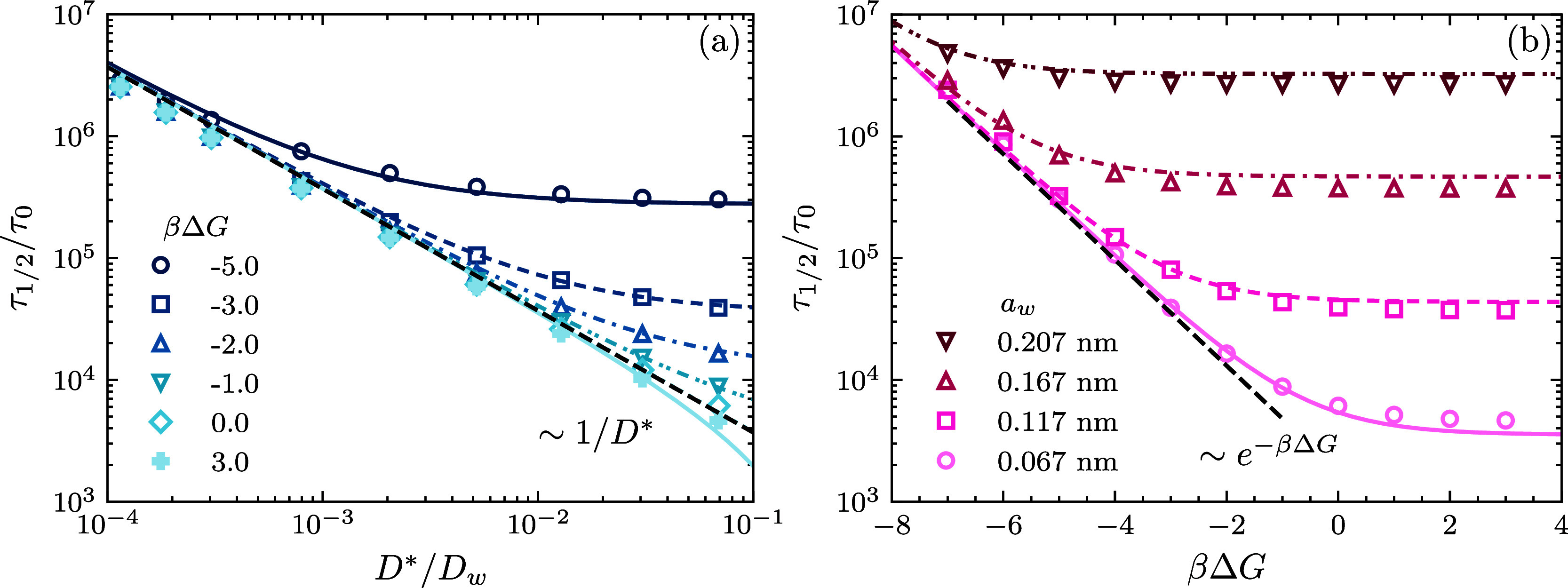
Normalized half-release time as a function of the diffusion coefficient and the molecule–microgel interaction free energy. Symbols represent results obtained from DDFT, while lines correspond to theoretical predictions from eqs 22 and 23. Figure 5a displays τ_1/2_/τ_0_ plotted against the diffusion coefficient inside the microgel D* normalized by the value in bulk, *D*_w_, for various values of the microgel–molecule interaction free energy, βΔ*G* = {−5,–3,–2,–1,0,3}. Conversely, Figure 5b illustrates τ_1/2_/τ_0_ as a function of βΔ*G* for different molecular sizes, given by *a*_w_ = {0.067,0.117,0.167,0.208} nm. For all presented cases, the molecule has a spherical geometry, with ρ_c_^0^ = 0.01 M, *b* = 100 nm, and δ = 1 nm.

As D* increases, indicative of smaller particle sizes, we observe a departure from the law τ_1/2_ ∼ 1/D* when examining attractive microgels. Indeed, beyond a certain point, the effects of negative Δ*G* over small cargo become significant in determining their release time. The reason for this relies on the fact that, for such fast molecules, the diffusive time inside the microgel becomes smaller. Therefore, the role played by the interaction free energy starts to be relevant, as the molecule needs to spend a significant time to overcome the step-edge free energy barrier at the microgel external surface in the case of attractive polymer networks. In other words, the molecule release becomes a reaction-limited process.

This is closely related to the dependence of the release time with the effective potential, shown as symbols in Figure 5b. When considering negative values of Δ*G*, we find that the release time increases as τ_1/2_ ∼ exp (−βΔ*G*) = exp (β |Δ*G*|) (see black dashed line), suggesting that the release kinetics follows the Arrhenius law. However, when the free energy is positive, its effects become negligible, indicating a saturation or threshold effect, beyond which the energetic profile exerted by the microgel does not affect the release time, leading to the diffusion-limited regime, where τ_1/2_ is independent of Δ*G*. In this realm of interactions, the τ_1/2_ ∼ 1/D* tendency is manifested through the logarithmic shifts for different molecular sizes (see eq 4).

Clearly, the existence of these two kinetic regimes indicates the presence of two major processes involved in the release kinetics: the diffusion through the microgel and the crossing of the free energy barrier. Later, we will delve into the role of these two contributions and propose an analytical model for τ_1/2_ in terms of both physical parameters.

It is important to remark that, as indicated by eqs 4 and 8, the values of D* and Δ*G* are not independent for a particular molecule. In other words, it is not possible to fix *a*_w_ (i.e., D*) and then arbitrarily vary Δ*G* and vice versa. In this sense, the plots in Figure 5 do not precisely represent results for a particular cargo molecule. Instead, they provide general physical insights useful for understanding the overall dependence of τ_1/2_ with our model parameters.

### 3.4 Effect of the Microgel Size

Figure 6 dives into the relationship between the half-release time τ_1/2_ and the radius of the collapsed microgel, *b*. The data obtained for different values of *a*_w_ and Δ*G* (shown as symbols) reveal that there is a direct proportionality between the release time and the square of the microgel’s radius, τ_1/2_ ∼ *b* ^2^. Clearly, a larger microgel requires a longer release time, since the encapsulated molecules have to diffuse a larger distance to reach the interface and escape to the bulk solution. This correlation is, in fact, related to a fundamental equation in diffusion studies known as the mean square displacement equation, ⟨Δ**r** ^2^⟩ = 6*Dt*. Interestingly, the square radius dependence observed for the characteristic release time of cargo molecules from dense microgels mirrors the same dependence reported for the characteristic swelling time of microgels in water.^69,70^ Both phenomena—cargo release and water molecule uptake—are governed by diffusion processes. As such, our findings are consistent with previously reported behaviors in diffusion-controlled systems.

**Figure 6:**
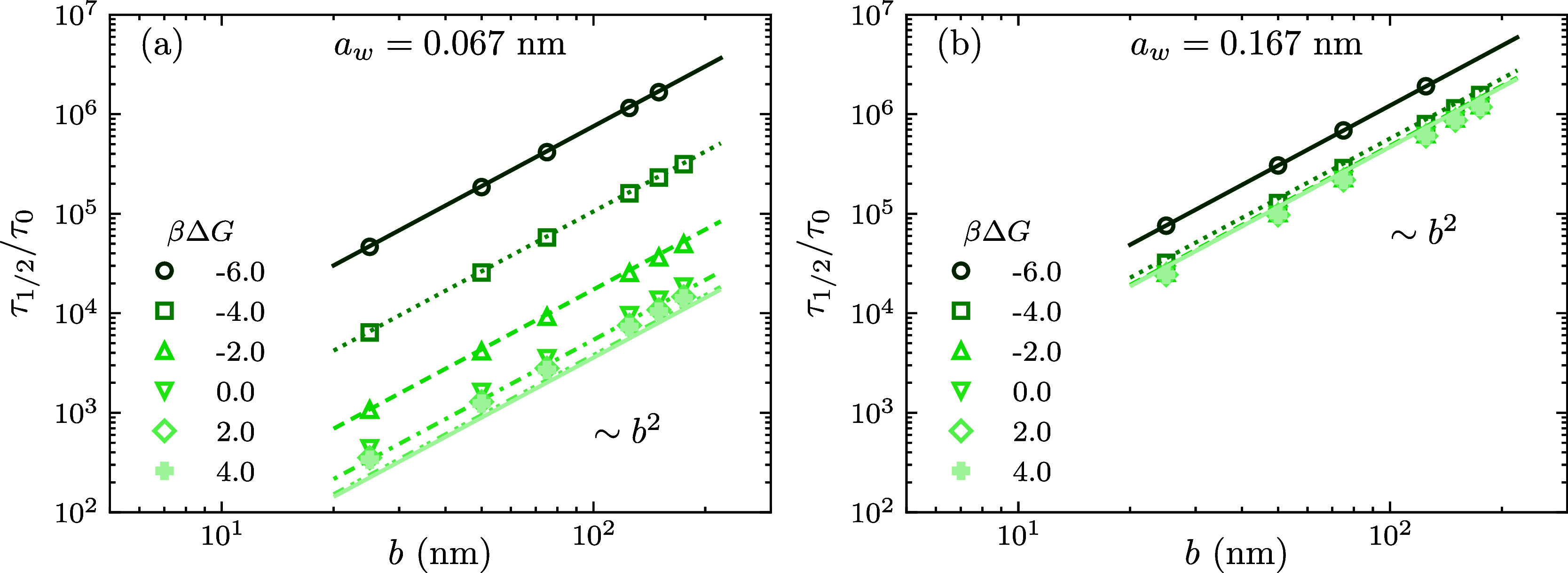
Half-release time, τ_1/2_, against the radius of the microgel *b*. The symbols represent data obtained by numerical integration of the DDFT equations, whereas the lines correspond to the theoretical predictions given by eqs 22 and 23. The left panel (Figure 6a) displays the case in which *a*_w_ = 0.067 nm, a value that is identified with neon. The right panel (Figure 6b) plots the same results but for *a*_w_ = 0.167 nm. Each plot shows results for several values of βΔ*G*, which extend from −6 to 4. In all cases, the geometry of the molecule is spherical, ρ_c_^0^ = 0.01 M, and δ = 1 nm.

We can also appreciate the effect that different molecular sizes and free energy potential depths have on the release time. Repulsive and noninteracting microgels (Δ*G* ≥ 0) behave in somewhat the same way: the transfer free energy has a very weak effect on the release time. However, for attractive microgels (Δ*G* < 0), the release time scales exponentially with the attraction strength, as observed in the log-scaled constant shifts of the release time in Figure 6a. In addition, Figure 6b shows that the influence that cargo size has over the kinetics is to universalize the release time all across the potential range once the molecular radii become sufficiently large. That is, diffusion through the microgel becomes a matter of size and not interaction. This is something that has been already noted in Figure 5a, where larger cargo shared a common τ_1/2_ regardless of the value of Δ*G*.

### 3.5 Analytical Expression for the Half-Release Time

In order to gather all the scaling trends into a unique analytical expression and provide a physical interpretation of the DDFT results, we calculate the mean-first passage time (MFPT), defined as the mean time that the molecules contained inside the microgel reach the bulk solution for the first time.^71–73^ Since the concentration of cargo molecules considered in our DDFT calculations is low everywhere, as a first approximation, the molecule–molecule interactions can be neglected, so the release process can be modeled as a diffusion problem of molecules through the effective potential induced by the microgel, *u*_eff_(*r*). In this limit, the DDFT equations simplify to the well-known Smoluchowski equation. We consider a suspension of microgels, wherein each microgel occupies, on average, a volume *V*. This volume *V* is the total system volume divided by the number of microgel particles. We approximate, as in our DDFT calculations, this volume *V* as a sphere of radius *R* such that the volume fraction of microgels in the suspension is φ = (*b*/*R*)^3^. We apply an absorbing boundary condition at *r* = *R*, which means that the molecules that reach this distance disappear from the system. We now turn our attention to a molecule initially located at a distance *r* = *s* from the center of the microgel. We are interested in determining the average time spent by this molecule to exit the designated volume, essentially reaching the surface of the spherical cell. The derivation, shown in detail in the Appendix, leads to the following expression for the MFPT^72^ ^,^ ^73^:

$$\overset{®}{\tau }(s)=\int_{s}^{R}dr\frac{e^{\beta u_{\mathrm{eff}}(r)}}{r^{2}D_{\mathrm{eff}}(r)}\int_{0}^{r}dr^{\prime }r^{\prime 2}e^{\beta u_{\mathrm{eff}}(r^{\prime })}$$ 19

In order to perform the integrals and obtain an analytical expression for τ̅(*s*), we approximate the interface of the microgel as a sharp boundary such that *u*_eff_(*r*) = Δ*G* for *r* ≤ *b* and *u*_eff_(*r*) = 0 for *r* > *b*. Analogously, the effective diffusion coefficient is simplified to *D*_eff_(*r*) = D* for *r* ≤ *b* and *D*_eff_(*r*) = *D*_w_ for *r* > *b*. Both assumptions are fully justified since collapsed microgels have a very narrow interface. With all these simplifications, we find the MFPT of a molecule located at *r* = *s* as

$$\overset{®}{\tau }(s)=\frac{b^{2}-s^{2}}{6D^{*}}+\frac{b^{3}}{3D_{w}}(\frac{1}{b}-\frac{1}{R})(e^{-\beta \Delta G}-1)+\frac{R^{2}-b^{2}}{6D_{w}}$$ 20

To calculate the average MFPT of all the encapsulated molecules, we have to perform a second averaging, accounting for the uniform distribution of molecules within the microgel in the initial stage, so τ̅ = ∫_0_^*b*^ *s* ^2^ τ̅(*s*)­d*s*/ ∫_0_^*b*^ *s* ^2^d*s*, leading to

$$\overset{®}{\tau }=\frac{b^{2}}{15D^{*}}+\frac{b^{3}}{3D_{w}}\left(\frac{1}{b}-\frac{1}{R}\right)\left(e^{-\beta \Delta G}-1\right)+\frac{R^{2}-b^{2}}{6D_{w}}$$ 21

Each term on the right-hand side of eq 21 has a clear interpretation. The first term is the average time of diffusion of the molecules inside the microgel to reach the microgel interface at *r* = *b*. The second term represents the time to overcome the free energy well Δ*G*. Finally, the third term is the time spent by the molecules to diffuse from *r* = *b* to reach the bulk at *r* = *R*.

For the comparison between this theoretical prediction and the DDFT calculations, we only need to retain the first two terms of eq 21, as they provide the time spent by the molecules to escape outside the microgel. Then, taking a very diluted suspension of microgels (*R* ≫ *b*), we obtain the MFPT for the molecules to escape from the microgel:

$$\overset{®}{\tau }=\frac{b^{2}}{15D^{*}}+\frac{b^{2}}{3D_{w}}(e^{-\beta \Delta G}-1)$$ 22

Finally, the corresponding half-release time will be given by

$$\tau_{1/2}=\overset{®}{\tau }/1.787$$ 23

Equation 22 gathers all the expected scalings of the release time with *b*, D*, and Δ*G*. Our equation aims to capture the intricate dynamics involved in the release kinetics of molecular cargo from collapsed microgels. This formula includes two terms that highlight the two fundamental forces at play. The first term mirrors the influence of pure diffusion on the release kinetics. This term embodies the classic principles of diffusive transport, suggesting that the larger the microgel, the longer the release time, while faster diffusion coefficients lead to shorter release times. The second term introduces the influence of the free energy profile of the microgel-cargo system.

As observed in Figures 5 and 6, the analytical estimate given by eq 22, represented by lines, demonstrates remarkable predictive accuracy across diverse conditions in our study. Particularly, this finding enables us to predict the release of cargo *f*_rel_(*t*) portrayed in Figure 4 by fusing eq 17 with eq 22. Consequently, the fraction of the released cargo can be accurately solved at any given time upon knowing the diffusion coefficient, the interaction microgel–molecule interaction free energy, and the radius of the microgel. We believe that this equation presents a powerful tool for researchers. It not only offers a conceptual framework for contemplating release kinetics but also provides a way for making preliminary predictions that can be refined using experimental data. We also believe that the equation can serve as a catalyst for further advancements in the field, fostering a more profound understanding of microgel behavior and potentially contributing to progress in applications such as drug delivery systems.

## 4 Conclusions

In this study, we have methodically elucidated the nonequilibrium diffusive release kinetics of small molecules from spherical microgel particles, extending our understanding from insights gained in previous all-atom simulation studies involving subnanometer-sized molecules within collapsed PNIPAM polymers in water. Molecular geometry emerged as a defining factor influencing the preferred directions of molecular motion, with linearly shaped molecules demonstrating more efficient transport through the microgel compared to their spherical counterparts. We employed DDFT to integrate the properties of transport molecules into a robust theoretical framework for evaluating the release kinetics from a spherical microgel particle. Notably, we have uncovered a universal behavior in the release dynamics of different molecules, characterized by a single parameter: the half-release time, τ_1/2_. This parameter incorporates all the essential system parameters: the diffusion coefficient within the polymer network, D*, and in water, *D*_w_, the interaction free energy of the molecule with the microgel, Δ*G*, as well as the microgel radius, *b*.

Finally, we have unified these key variables into a single analytical expression for τ_1/2_ (given by eq 22), which has demonstrated excellent agreement with our DDFT calculations. The formula provides a comprehensive understanding of the physics governing the release kinetics. The release process manifests into two distinct limiting regimes: for large, slowly diffusing, and poorly soluble molecules within the hydrogel (exhibiting large Δ*G*), the diffusion-limited regime prevails, where the release time inversely scales with their diffusion coefficient within the gel (τ_1/2_ ∼ *b* ^2^/D*). Conversely, small, rapidly diffusing, and highly soluble molecules (with small or negative Δ*G*) tend toward the reaction-limited regime. In this scenario, the bottleneck becomes the overcoming of the free-energy step-edge barrier to escape the microgel, resulting in the release time scaling exponentially with the free energy (τ_1/2_ ∼ *b* ^2^ exp (−Δ*G*/*k*_B_ *T*)). In both cases, the relationship between the microgel particle size and τ_1/2_ strictly adheres to a well-known power law (τ_1/2_ ∼ *b* ^2^).

To the best of our knowledge, the simple mathematical expression for the release time (eq 22) has not been previously reported in the literature. Notably, the theoretical framework we employed is applicable not only to microgels but also to a wider range of hydrogel structures and other porous materials. Assuming that the diffusion coefficient and the interaction free energy between the molecule and the polymer network are known, the diffusion-solution principles at the macroscopic level remain applicable. While we specifically designed the model for PNIPAM microgels in their collapsed state, our theory has a broader scope, particularly for particles with clearly defined and sharp interfaces.

However, it is important to note the limitations of our current model. In this regard, while the absorbing boundary condition is appropriate for very dilute solutions, we acknowledge that for more concentrated suspensions and longer times, reflective boundaries are more accurate. Indeed, for a nondilute suspension of microgel particles, the final equilibrium concentration of cargo molecules in bulk tends to a nonzero constant value, which is achieved by imposing reflective boundary conditions at the external boundary of the spherical cell (*r* = *R*). In this case, the release kinetics becomes strongly dependent on the microgel volume fraction, adding an additional parameter to consider in the analysis. As many experiments and applications of drug release are performed under highly diluted conditions, the correction introduced by the reflective boundary conditions only affects the very late stages of the release process, leaving the estimate of the half-release time almost unaffected. The study of release kinetics in concentrated microgel suspensions is out of the scope of this paper and will be part of our future work.

Despite this limitation, our research provides valuable insights and establishes a foundation for further studies. We aim to explore the release kinetics from swollen microgels in our future work, anticipating differences in diffusion compared to collapsed states. Ultimately, our goal is to develop a comprehensive theoretical framework capable of predicting cargo diffusion within microgel particles in various swelling conditions.

## Appendix. Calculation of the mean first passage time

In the limit of negligible molecule–molecule interactions, the DDFT formalism converges to the Smoluchowski equation for the time-dependent probability density, ρ­(**r**, *t*), defined as the probability density of finding a Brownian particle (molecule) at position **r** at time *t* ^72,73^

$$\frac{\partial \rho }{\partial t}=\nabla \cdot \left[D\left(r\right)\nabla \rho +D\left(r\right)\rho \beta \nabla u\left(r\right)\right]$$
24

where *D*(**r**) is the position-dependent diffusion coefficient, and *u*(**r**) is the external interaction potential acting on the Brownian molecules. If at time *t* = 0, the molecules are localized in a very small region at position **r**, the probability density can be denoted as ρ­(**r** ^′^, *t* | **r**,0), which satisfies the initial condition ρ­(**r** ^′^,0 | **r**,0) = δ­(**r** – **r** ^′^).

We assume that the molecules are contained within a volume *V*. The molecules can escape from this volume by reaching its surface Σ. This means that the system has an absorbing surface, with ρ­(**r** ∈ Σ, *t*). The probability that a given molecule initially located at position **r** inside *V* at *t* = 0 escapes outside this volume at time *t* will be denoted by *W*(*r*, *t*), given by

$$W(r,t)=1-\int_{V}\rho (r^{\prime },t|r,0)dr^{\prime }$$
25

This new function *W* satisfies several conditions. On the one hand, if the point **r** is located at the surface of *V*, then *W*(**r**, *t*) = 1. On the other hand, if **r** is a point inside of *V*, then *W*(**r**,0) = 0 and *W*(**r**, ∞) = 1. *W*(**r**, *t*) obeys the following partial differential equation^72,73^ :

$$\frac{\partial W}{\partial t}=\nabla \cdot [D(r)\nabla W]-D(r)\beta \nabla u(r)\cdot \nabla W$$
26

The probability that a molecule located at **r** at time *t* = 0 arrives at the surface of the volume and escapes outside between *t* and *t* + d*t* is

$$W(r,t+dt)-W(r,t)=\frac{\partial W(r,t)}{\partial t}dt$$
27

Therefore, the average time for a molecule located at **r** at time *t* = 0 to arrive at the surface is given by the following integral,

$$\overset{®}{\tau }(r)=\int_{0}^{\infty }t\frac{\partial W(r,t)}{\partial t}dt$$
28

This is precisely the so-called mean-first passage time (MFPT). We can deduce the differential equation that satisfies the MFPT by applying a second-time derivative in eq 26, multiplying by *t* and then integrating over *t*:

$$\int_{0}^{\infty }t\frac{\partial^{2}W}{\partial t^{2}}dt=\nabla \cdot [D(r)\nabla \overset{®}{\tau }(r)]-D(r)\beta \nabla u(r)\cdot \nabla \overset{®}{\tau }(r)$$
29

Integrating by parts the left-hand side of this equation leads to

∫0∞t∂2W∂t2dt=t∂W∂t|0∞−∫0∞∂W∂tdt=W(r,0)−W(r,∞)=−1
30

The resulting differential equation for the MFPT is

$$\nabla \cdot [D(r)\nabla \overset{®}{\tau }(r)]-D(r)\beta \nabla u(r)\cdot \nabla \overset{®}{\tau }(r)=-1$$
31

Rewriting this equation to the particular case of the spherical geometry, with the volume *V* representing a sphere of radius *R*, we obtain

$$\frac{1}{r^{2}}\frac{d}{dr}\left[r^{2}D(r)\frac{d\overset{®}{\tau }}{dr}\right]-D(r)\frac{d(\beta u)}{dr}\frac{d\overset{®}{\tau }}{dr}=-1$$
32

which can be expressed as

$$\frac{d}{dr}\left[r^{2}D(r)e^{-\beta u(r)}\frac{d\overset{®}{\tau }}{dr}\right]=-r^{2}e^{-\beta u(r)}$$
33

This differential equation can be integrated analytically, using the fact that the MFPT is finite for any point within the volume *V* and equals zero if the molecule is located at the external surface of radius *R*, τ̅(*r* = *R*) = 0. Performing the first integration gives

$$\frac{d\overset{®}{\tau }}{dr}=-\frac{e^{\beta u(r)}}{r^{2}D(r)}\int_{0}^{r}dr^{\prime }r^{\prime 2}e^{-\beta u(r^{\prime })}$$
34

Finally, the MFPT for a molecule located at a distance *r* = *s* from the center of spherical volume is obtained through the second integration

$$\overset{®}{\tau }(s)=\int_{s}^{R}dr\frac{e^{\beta u(r)}}{r^{2}D(r)}\int_{0}^{r}r^{\prime 2}e^{-\beta u(r^{\prime })}dr^{\prime }$$
35
